# Soldiers and the Provincial Hospitals

**Published:** 1914-10-10

**Authors:** 


### Soldiers and the Provincial Hospitals.

### BATH.

The Royal Mineral Water Hospital has received from the War Office an acknowledgment of its patriotic offer. The reply states that, although the beds are not required at the present moment, a few suitable patients will be sent there from time to time. The Mayoress of Bath's Needlework Guild has forwarded to the local Red Cross Branch sufficient garments, etc., to supply a hospital of 100 beds, and in addition to garments sent to soldiers and the Central Red Cross Depôt in London, boxes have been forwarded direct to the Rouen Base Hospital and Rouge Croix Français. The baths committee of the Bath Bathing Establishment have offered the free use of any of the mineral water or other baths to sailors and soldiers likely to benefit from such treatment.

### BRISTOL: ARRIVAL OF SERIOUS CASES.

Further contingents of wounded continue to arrive at the 2nd Southern Hospital at Bristol. The 300 recent cases were for the most part of a much more serious nature, necessitating a considerable amount of surgical work. One hundred of those who arrived were allocated to the Southmead Infirmary, and it gives some idea of the excellent organisation when it is stated that every patient of the above 300 was in bed, washed, and had received a first dressing within three hours of his arrival at Bristol.

---

The "F" Bearer Company of the City of Bristol Corps of the St. John Ambulance Association left Bristol on Saturday, September 26, for Southampton *en route* for Paris, where they are to act as the personnel attached to the Duchess of Westminster's War Hospital of 200 beds. Previous to their departure the detachment, consisting of fifty-six men, was inspected at the headquarters, when the Duke and Duchess of Beaufort and the district Deputy Commissioner, Dr. J. S. Griffiths, were present. On the following Monday a large number of the Bristol Guardians paid a visit to the Southmead Infirmary, where they were received by Colonel Bush, who, after a complete inspection of the institution had been made, expressed gratitude to the Guardians for their generosity in placing the infirmary at the disposal of the War Office. The number of patients able to be received at the Royal Infirmary and at the Southmead institution is 1,040, and 550 beds are now ready at the latter, where *x*-ray apparatus has also recently been installed. The Mayor of Bristol, who was prevented from visiting the Southmead Infirmary with the Guardians, has since taken the opportunity of doing so.

### CAMBRIDGE: UNLOADING A HOSPITAL TRAIN.

From the many thousands of British soldiers who encamped in and around Cambridge under canvas, about 130 who have been wounded in action returned to Cambridge on Thursday, last week. About thirty of the wounded were unable to walk, and were removed from the train on stretchers. There were among the wounded men of the Norfolk, Suffolk, East and West Yorks Regiments, Royal Fusiliers, and Royal Horse Artillery, many of whom only left Cambridge a few weeks since.

The authorities at the 1st Eastern General Hospital, under the command of Colonel J. Griffiths, M.D., M.C., F.R.C.S., were notified on September 22 of the fact that about 194 of our soldiers who had been wounded in action were on their way to Cambridge, and in all probability would arrive about midnight. Without delay the sick and wounded already in the hospital at Trinity College as many as could without harm be moved from the wards were transferred to canvas accommodation.

The exact time of arrival of the wounded was not known; nevertheless sufficient stretcher-bearers of the local Voluntary Aid Detachments were in readiness at Cambridge Station by 11 p.m. A large number of ladies of the Shelford and Harston Voluntary Aid Detachments, in uniform, prepared refreshments ready for those of the wounded who were able to take them. By midnight the station yard became a centre of great activity, yet perfect order. The Rev. C. F. Townley, who has spared neither time, money, nor energy in connection with the work of the local branch of the British Red Cross Society, was present at the station in command of the stretcher-bearers.

The first train steamed into the station about 1 a.m., on Wednesday. To those acquainted with hospitals and hospital work the train was a travelling hospital. There were not only compartments representing wards, but every one of the various administrative departments, from the office downwards, equipped with telephone and typewriter, etc. Every arrangement had been made in this travelling hospital for the comfort of the wounded.

The stretcher-bearers, receiving their orders from the officers in charge of the train, quickly and quietly transferred the wounded from the train to the R.A.M.C. ambulance and other conveyances waiting in the station yard, and they were rapidly conveyed to the wards at the 1st Eastern General Hospital at Trinity College. This piece of work had scarcely been completed when the second Red Cross hospital train arrived on the Great Eastern platform with a second batch of wounded troops, and although seriously wounded many were able, to use their own expression, "to shift for themselves," and they in their turn were conveyed to the hospital, where everything was ready for their reception.

A number of these wounded soldiers are being subjected to $x$-rays in the electrical and $x$-ray department at Addenbrooke's Hospital, Cambridge, which department is under the care of Dr. F. Shillington Scales.

The erection of the new 1st Eastern General Hospital on Clare College cricket ground, to accommodate at least 500 wounded soldiers, is progressing with great rapidity.

### CARDIFF: A WOUNDED SOLDIER'S THANKS.

No fresh cases from the Front have arrived at Cardiff up to the time of writing. The discharges to date number 110. The work of equipment proceeds apace, and a further large quantity of beds are in readiness.

The following letter has been sent to the Cardiff *Western Mail*:—"Please allow me through the medium of your interesting paper to convey to the staff and nursing sisters at Albany Road (Cardiff) Schools my heartfelt thanks for the kindness and attention enjoyed by me during the time I was there as an invalid. Many of my comrades will join me, I know, in praising their untiring efforts for our welfare. My sojourn there I shall always recall with pleasure. Allow me also to thank the public for their hospitality and kind regard. My thanks to all is heartfelt and sincere.—B. W. Owen, Argyll and Sutherland Highlanders, Bath. October 3, 1914."

The Glamorgan Depôt of the Red Cross Society is located at Cardiff under the superintendency of Miss Perry, who is doing admirable work in co-ordinating the various Societies engaged in making articles of clothing and other requisites for wounded sailors and soldiers. An enormous quantity of gifts, including fruit, eggs, jam, etc., has been received and distributed to no fewer than the following five organisations: The Base Hospital, the Welsh Hospital, the Welsh Horse, the Soldiers' and Sailors' Association, Queen Mary's Needlework Guild.

### CHATHAM DISTRICT: KING AND QUEEN'S VISIT.

Their Majesties the King and Queen paid a visit to Chatham last Saturday, and, after lunching at Government House, visited the wounded sailors at the Royal Naval Hospital and the wounded soldiers at the military hospital at Fort Pitt. The townspeople turned out in thousands, and gave their Majesties an enthusiastic welcome.

Wounded soldiers have been transferred from the Fort Pitt Hospital to St. Bartholomew's Hospital, Rochester. They are special cases. The Strood Voluntary Aid Detachment's Hospital have now sixty wounded soldiers under their charge. Their hospital at Claremont House is full, and fifteen men are being accommodated in the Co-operative Hall. Most of the patients are removals from Fort Pitt Military Hospital or the Drill Hall close by, but several have been received direct from the Front, one wearing pyjamas in lieu of trousers, and another German boots with spurs attached.

### GRAVESEND AND DISTRICT: SOLDIERS' TALES.

The first batch of wounded arrived at the Gravesend Hospital on September 29 and came through the Central Military Hospital at Chatham. They are representative of the various British regiments, and are mostly suffering from shrapnel bullet-wounds received at the Battle of the Aisne. Motor-cars conveyed them to the hospital, and the men appreciated the ease and comfort of the transport. A list has been compiled of private owners who are willing to lend their cars for the conveyance of wounded from the ambulance-trains to the various hospitals in the district. When wounded are expected the owners are informed, and the cars are there waiting when the train arrives. The soldiers are full of stories, but they much prefer to talk to their friends of the good qualities of the hospital cook and of the kind and attentive treatment they are receiving than of their exploits in the field.

### LEICESTER: A THIRD BATCH OF WOUNDED.

The arrival of a third ambulance train from Southampton on Thursday, last week, with 130 wounded soldiers has brought the number of patients in the 5th Northern General Hospital to over 300. The larger proportion of the last arrivals suffered from injuries of a serious nature, chiefly sustained in the battles on the Aisne and the Marne. The wounded comprised many non-commissioned officers and men who had been with the Expeditionary Forces from their arrival in France and had fought through the engagements at Mons and Charleroi, and they brought thrilling tales of the terrible time they had experienced. All bore testimony to the medical care they had received, and to the excellence of the food supply to the entire British Force. The ambulance train was met by Mr. A. W. Faire, County Director Voluntary Aid Detachments, and his assistant, Mr. W. F. Seymour, the medical service at the station was in charge of Lieut.-Col. Pemberton Peake, and the nursing service under the supervision of Miss Pollard. A fleet of motor-cars, forty in number, was in charge of Mr. J. Reginald Corah, and speedily conveyed the men from the station to the hospital, before a large number of sympathetic spectators. On arrival at the hospital the men were all medically examined under the supervision of Lieut.-Col. Harrison, officer in command, and conveyed to their respective wards without delay. It is pleasing to report that all are progressing as well as can be expected. To meet the increased demands on the medical and nursing services, Major T. V. Crosby and Captain R. H. Fagge have been mobilised and further nurses called up.

A first death has been reported during the week from tetanus, after a wound caused by shrapnel. The medical officers intimate that their experience of shrapnel wounds is that they almost invariably grow septic, and are slow of healing; bullet wounds, on the contrary, being generally clean and healing readily under medical treatment. It is understood that instructions have recently been received from headquarters that the base hospital is to be reserved for cases from the Expeditionary Forces; those from Territorial camps are to be provided for elsewhere. This probably indicates the utilisation of private houses, many of which have been offered to the County Director. A pleasing feature in connection with the hospital during the week has been the presentation by the Mayor and Mayoress (Councillor J. R. and Mrs. Russell Frears) of framed steel engravings of the King and Queen for the men's dining-room. The gift was accepted by the officer in command (Lieut.-Col. Harrison) in the presence of a large number of patients and the staff of the hospital. Colonel Harrison eulogised the great service which the Mayor had rendered in connection with the Prince of Wales' Fund and other efforts, and the energy of the Mayoress in directing the efforts of the people of Leicester in the provision of useful articles for the comfort of the soldiers, with the result that 500 flannel shirts and many under-garments had arrived at the hospital. Major R. W. Henry (the Registrar) thanked the donors for their kindness and their thoughtfulness. A temporary chapel is in course of construction, and it is hoped will be in readiness for dedication by St. Luke's Day (October 18). The series of entertainments for the patients twice weekly has been continued, to the great enjoyment of the patients and staff.

### SHEFFIELD: THE THIRD CONVOY.

The third convoy of wounded arrived in Sheffield last Saturday afternoon for treatment at the 3rd Northern General Hospital. The percentage of wounded is considerably higher than previously, there being thirty-five stretcher cases out of 120. The convoy came from Southampton on a hospital train, and reached Sheffield Midland Station at a quarter to four on Saturday afternoon. It was expected the previous night, but the transport had been delayed in the Channel by fog. The convoy was met by stretcher parties from the local unit of the R.A.M.C. and from the Sheffield Fire Station. Under the direction of Colonel Connell and Captain Pooley the wounded soldiers were skilfully and swiftly transformed in relays from the train to two Corporation motor-buses and six ambulances.

The arrival of this new convoy brings up the total number of wounded soldiers in the hospital to nearly three hundred. In addition, a number of Territorials are being treated for various injuries and ailments. Of the first convoy all save eight men have been discharged, and of the second convoy all save eighty-four.

The work of the hospital has been principally surgical. An enormous percentage of the cases have been *x*-rayed for the removal of pieces of shrapnel and bullets. Through the kindness of the local manager of the Empire Music Hall in the city, thirteen variety artistes gave a delightful entertainment to the patients on Friday afternoon. Colonel Connell would also like to say how much they appreciate the various gifts of fruit and game.

The Countess Fitzwilliam has presented the hospital with an ambulance trolley, and Mrs. Gertrude Wilson has also sent a cheque for £15 for any purpose Colonel Connell thinks fit, and he has found it most valuable for essentials not included in the scale of equipment laid down, but which are found useful in civilian hospitals.

### THE FIRST WOUNDED AT TUNBRIDGE WELLS.

The first wounded from the Front arrived in Tunbridge Wells for treatment at the General Hospital on Tuesday last at about 4 p.m. The men were conveyed from Chatham in private motor-cars lent by the following residents:—Sir David L. Salomons, Bart., Mr. E. Tindall, Mr. A. Le Blond, Mrs. Beer, Mr. C. W. Hay, Mr. A. H. Massey-Cooke, Mr. C. C. Jury, Mr. D. Elliott Alves, Miss Thornton, Mr. J. S. Sword, and Mr. Hamilton Gordon. The men were given an extremely warm welcome by a very large crowd.

A contingent of the St. John Ambulance Brigade, Dr. Ballantine, the house physician, together with Kemp, the head porter, and Carson, the second porter of the General Hospital, were with the cars to see that all was well with the wounded during their journey from Chatham. Within fifteen minutes from the arrival of the first car all the men (twenty-four) were safely in the ward. Most of the wounds were inflicted at the Battle of the Aisne by shrapnel. All are making good progress, with the exception of one poor fellow, who was shot in the foot over three weeks ago, and on Saturday last tetanus developed. So far progress is satisfactory.

